# Development of an Integrated Radiotherapy Simulation Platform with AI-Driven Segmentation and Ray-Casting-Based Dosimetric Evaluation

**DOI:** 10.3390/bioengineering13050572

**Published:** 2026-05-18

**Authors:** Cheng-Yen Lee, Hsiao-Ju Fu, Pin-Yi Chiang, Hien Vu-Dinh, Hung-Ching Chang, Hong-Tzong Yau

**Affiliations:** 1 Department of Mechanical Engineering, National Chung Cheng University, Chiayi 621301, Taiwan; 07463@cych.org.tw (C.-Y.L.); 10260@cych.org.tw (H.-J.F.); vuhien260697@alum.ccu.edu.tw (H.V.-D.); sujieghxb@gmail.com (H.-C.C.); 2 Advanced Institute of Manufacturing with High-Tech Innovations, National Chung Cheng University, Chiayi 621301, Taiwan; 3 Department of Radiation Oncology, Ditmanson Medical Foundation Chia-Yi Christian Hospital, Chiayi 600566, Taiwan; 4 Ph.D. Program of Interdisciplinary Medicine, National Yang Ming Chiao Tung University, Taipei City 112304, Taiwan

**Keywords:** point transformer, Percentage Depth Dose (PDD), radiation treatment planning, virtual radiotherapy simulation

## Abstract

Radiotherapy simulation is essential for accurately targeting tumors while preserving healthy tissue, ensuring treatment precision and safety. This study aimed to develop an integrated radiotherapy simulation system capable of automated segmentation, dose estimation, and collision detection within a virtual planning environment to enhance efficiency and reduce costs in radiotherapy treatment planning. The Point Transformer model was applied to organ point cloud data derived from CT medical imaging for automated segmentation. Farthest point sampling (FPS) was employed to downsample the data before training. To enhance the accuracy and anatomical fidelity of the AI-generated segmentation results, reconstruction and refinement algorithms, including k-d tree, outlier removal, marching cubes, and surface smoothing, were implemented. Beam penetration simulation with the ray casting algorithm was employed for correction-based dose estimation. A collision detection module was incorporated to identify potential machine–machine or machine–patient interactions. The entire workflow was executed within a Unity 3D-based virtual simulation environment. As a result, the Point Transformer model demonstrated high segmentation accuracy, achieving Dice scores of 93.86 ± 1.50% for single-organ and 91.86 ± 3.25% for multi-organ cases, surpassing the performance of PointNet++. Applying ray casting for the refined surface meshes generated through post-processing enabled accurate dose estimation with discrepancies of 3.5% (brain), 5.9% (liver), and 13.8% (lung) compared to a Pinnacle TPS. The proposed method provides a low-cost and adaptable solution that enables easy modification and further development, making it particularly suitable for widespread applications in radiotherapy research, education, and clinical workflow optimization.

## 1. Introduction

Radiotherapy treatment planning has developed rapidly over the past century [1,2,3]. From the early 20th century, treatment plans were created manually using 2D patient contours and standard isodose charts derived from measured percentage depth-dose (PDD) data [1]. By 1972, the invention of X-ray computed tomography (CT) by Hounsfield and Cormack, along with the computer-assisted calculations, enabled true 3D planning [2,3]. For the first time, CT scanning allowed 3D visualization of the tumor and normal organs, permitting accurate calculation and optimization of dose distribution in the target volume while protecting surrounding tissues. In the 1980s, three-dimensional conformal radiotherapy (3D-CRT) was developed to design beam configurations using the beam’s eye view visualization [4]. Treatment plans could be evaluated with dose uniformity overlays and dose–volume histograms (DVHs). Since the 1990s, the concepts of inverse planning using algorithms to optimize intensity-modulated radiotherapy (IMRT) and volumetric modulated arc radiotherapy (VMAT) have been introduced [5]. These techniques enabled conformal dose sculpting by automatically optimizing small beamlets with computer algorithms, rather than relying on manual beam arrangement trials. As a result, radiation could be delivered with millimeter-level accuracy, and this approach remains the standard of practice today. Over time, radiation therapy simulation has evolved from a purely geometric setup into a comprehensive computerized process that includes imaging, contouring, dose calculation, and treatment plan optimization.

Imaging is fundamental to radiotherapy simulation because it enables accurate identification of patient anatomy for precise treatment planning [3,6,7]. CT remains the most widely used modality because it provides reliable geometric information in most cases. In clinical practice, additional imaging modalities, such as magnetic resonance imaging (MRI) and positron emission tomography (PET), are often combined with CT to address the limitations of any single technique and to improve the delineation of complex anatomical relationships [8].

Once imaging datasets are acquired, the next essential step is delineation of target and organ at risk (OARs) to guide dose optimization and minimize normal-tissue toxicity. Contouring techniques have undergone tremendous development from the manual, wire-based body-outline tracings of the pre-CT era to the semi-automated and fully automated methods available today [9,10]. The introduction of CT has increased segmentation accuracy compared to earlier approaches, which involved tracing the patient’s body contour using mechanical aids such as flexible leads or solder wires to capture transverse outlines. However, manual delineation on CT remains a labor-intensive and time-consuming process, often requiring several hours for complete segmentation of all relevant structures [11]. Moreover, inconsistency between operations has always been an issue [12]. Therefore, early auto-contouring algorithms were developed, such as simple threshold-based methods [13], nearest-point line approaches [14], and atlas-based segmentation [10]. While successful for specific organs, these methods have encountered limitations in accuracy, generalizability across different anatomical regions, and fully automated performance. In recent years, artificial intelligence (AI) and deep learning approaches have transformed the contouring process [9,15,16]. Advanced neural network models trained on large datasets of expert-annotated scans are now capable of automatically delineating organs [17]. The integration of deep learning has significantly improved the quality and accessibility of auto-segmentation, producing contours that are far more accurate and consistent than earlier methods [18].

The primary objective of radiotherapy is to accurately deliver the prescribed dose to tumor tissues while minimizing exposure to surrounding healthy organs. Therefore, dose calculation plays a critical role in radiotherapy (RT) treatment planning [19,20]. The development of dose calculation has progressed from empirical approaches to sophisticated physics-based algorithms [21,22]. Early treatment planning systems (TPS) relied on measured beam data, such as percentage depth-dose (PDD) curves and tissue-air ratios (TAR), to manually estimate the dose at specific points, typically referencing only the central-axis depth [23]. Although these methods were simple, fast, and effective for cases involving flat and homogeneous tissue regions, they lacked the accuracy required for evaluating 3D dose distributions and tissue heterogeneities. The introduction of CT imaging, which provides a 3D patient-specific electron density map, greatly advanced the development of computerized dose calculation algorithms [20,24]. These developments included pencil-beam models and Clarkson integration techniques in the 1980s, followed by convolution/superposition algorithms in the 1990s. Around the same time, Monte Carlo (MC) simulation became the gold standard for dose calculation because of its high accuracy in modeling dose deposition. However, its clinical use was initially limited by high computational demands. Recent advances have improved calculation speed, making MC methods more practical. Overall, the choice of dose calculation method depends on the clinical scenario and must balance accuracy with computational efficiency [21,25].

Modern radiotherapy platforms now incorporate a wide range of advanced functionalities to improve treatment safety and streamline clinical workflows [26]. Beyond the core tasks of imaging, delineating organs, and calculating dose distributions, current systems also offer capabilities in sophisticated plan optimization, adaptive replanning, and data-driven decision support. However, these functions are often proprietary products of a few large vendors. The closed design and high computational cost can restrict access and hinder customization [27,28].

In this study, we developed a radiotherapy simulation system that enables automated segmentation, radiation dose estimation, and collision detection during treatment planning. The Point Transformer neural network model was implemented to segment OARs from CT medical imaging. To reduce training cost, the farthest point sampling (FPS) technique was applied on the input data. The AI-generated segmentation results were refined using post-processing algorithms, including k-d tree construction, outlier removal, marching cubes, and surface smoothing, to enhance the accuracy and anatomical fidelity of the segmented organs. Dose estimation at the tumor center can be performed in sync with beam selection, which is a foundational step in 3D-CRT and IMRT planning. Beam selection defines the geometric search space for optimization. In clinical planning, this process is primarily guided by the Beam’s Eye View (BEV), which helps identify beam angles that maximize target coverage while minimizing overlap with critical organs. Additionally, a collision detection module, based on a depth camera and the separating axis theorem algorithm, was integrated to evaluate potential interactions between the radiotherapy machine and the patient. All functionalities were implemented and simulated within a Unity 3D virtual environment to support precise and effective radiotherapy planning.

## 2. Materials and Methods

**Figure 1:**
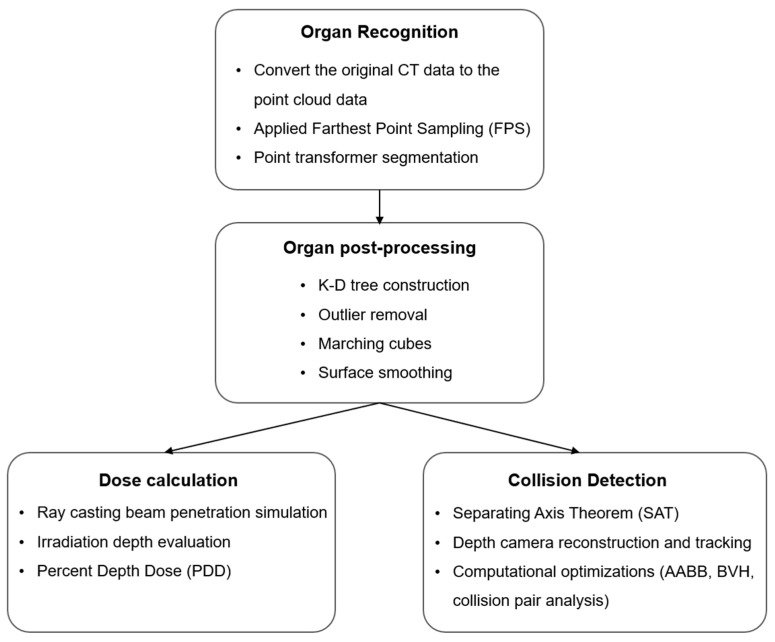
Research workflow.

This study was reviewed and approved by the Institutional Review Board of Chiayi Christian Hospital (IRB2025011). Manually segmented CT images from patients who had undergone radiotherapy were employed. A radiotherapy simulation system was developed in a Unity 3D virtual environment. Its functions included automated organ segmentation, radiation dose estimation, and collision detection during treatment planning (Figure 1).

The software environment consists of Python 3.9.12 with PyTorch 1.10.2 for deep learning implementation, CUDA 11.x for GPU acceleration, and supporting libraries including SimpleITK and PyVista for medical image and point cloud processing, as well as NumPy, SciPy, and scikit-learn. The virtual simulation environment was developed using Unity 3D (version 2020.3.19f1). All experiments were conducted on a workstation equipped with an Intel^®^ Core™ i7-9700KF CPU (3.60 GHz, 8 cores), an NVIDIA Tesla P40 GPU with 24 GB VRAM, and 31 GB of system memory (2666 MT/s).

### 2.1. Automated Image Segmentation for Organ Recognition and 3D Surface Reconstruction

Point Transformer [15] was employed for organ recognition using the WORD dataset [29], which provides annotated organ contours as ground truth. Two evaluation scenarios were considered: single-organ recognition (liver) and multi-organ recognition (liver and spleen). For training, the 3D volumetric CT scan data (.nii.gz) were converted into point-cloud-like sequential data (.*xyz* coordinates, HU values, and labels). Specifically, SimpleITK [30] was used to extract the origin and voxel spacing, while PyVista [31] mapped voxel indices to physical coordinates (*x*, *y*, *z*). A dual-condition filtering strategy was applied to retain voxels that either belong to regions of interest (ROIs; predefined target labels) or fall within a specified HU range. Finally, label masking was performed to assign non-target voxels to the background (label 0), and the data were flattened into 1D arrays (coordinates, HU, labels). This process reduces background redundancy while preserving essential spatial and density information for downstream modeling.

To balance computational efficiency and cost, farthest point sampling (FPS) [32] was applied to downsample each point cloud to a uniform size of 32,000 points per case. These processed point clouds were then used as input to the Point Transformer model, which predicts the label information for each point.

The point transformer architecture builds on self-attention mechanisms designed for irregular and unordered 3D data. This design enables the model to effectively capture local geometric structures and long-range dependencies. The model consists of an encoder–decoder structure with TransitionDown and TransitionUp modules for hierarchical feature extraction and reconstruction, respectively. Each stage of the encoder integrates a PointTransformerLayer, which applies spatially aware attention across neighboring points using learned positional encodings and attention weights. The decoder progressively upsamples features while fusing information from corresponding encoder layers, allowing fine-grained semantic predictions. This implementation provides multiple variants (Seg26, Seg38, Seg50) with varying depths, enabling trade-offs between performance and computational cost.

The dataset was partitioned into training (100 samples), validation (20 samples), and test (30 samples) subsets. The predicted results were compared with the ground truth annotations to evaluate model accuracy. In addition, a comparative analysis of prediction performance and visualized point clouds was conducted against PointNet++ [33] using the same dataset.

The raw outputs obtained by Point Transformer require further processing before being used in dose estimation. During both training and inference, point cloud data undergo sampling, which leads to a loss of fine surface details. To recover these missing features, a k-d tree-based nearest neighbor search algorithm [34] was employed to propagate labels from the downsampled predictions back to the original high-resolution point cloud. After that, a statistical outlier removal technique was applied to eliminate noisy points near the liver boundaries. For downstream applications, such as dose evaluation and radiation-object collision detection, pointwise operations are essential. However, performing these calculations directly on large-scale point clouds is computationally expensive and often insufficient for fully representing complex organ geometries. To overcome this, the labeled point clouds were voxelized and converted into surface meshes using the marching cubes algorithm [35]. Finally, Laplacian smoothing [36] was applied to enhance surface fidelity as the marching cubes-reconstructed meshes are typically coarse.

### 2.2. Dose Evaluation

**Figure 2:**
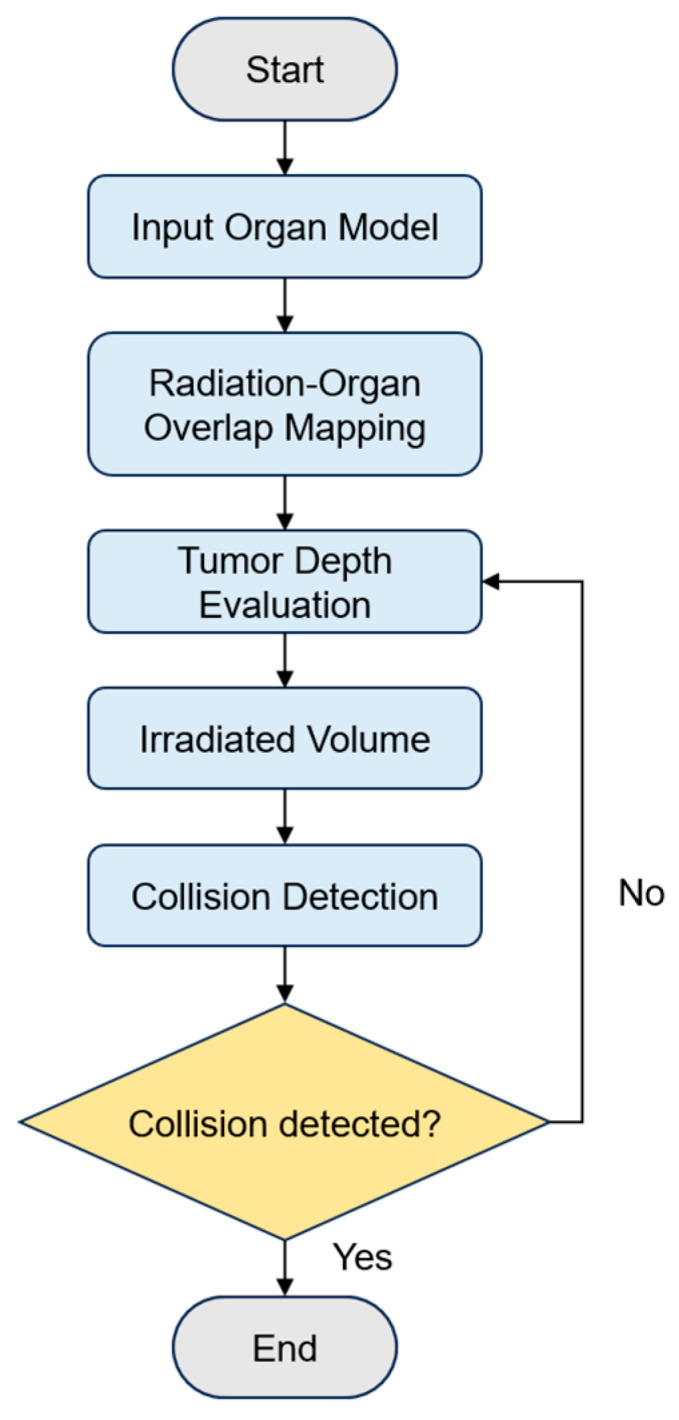
Workflow for radiotherapy treatment planning optimization.

The organ point cloud data extracted through Point Transformer were converted into surface triangular meshes in OBJ format. The ray casting method was employed to simulate interactions between radiation beams and organs. This process identifies the entry and exit points of radiation beams across the triangular meshes, enabling the calculation of the path length traversed within each organ. Based on this information, the absorbed dose delivered to the tumor was estimated. This process supports the optimization of radiotherapy treatment planning, as illustrated in Figure 2.

#### 2.2.1. Ray Casting for Intersection and Beam Path

**Figure 3:**
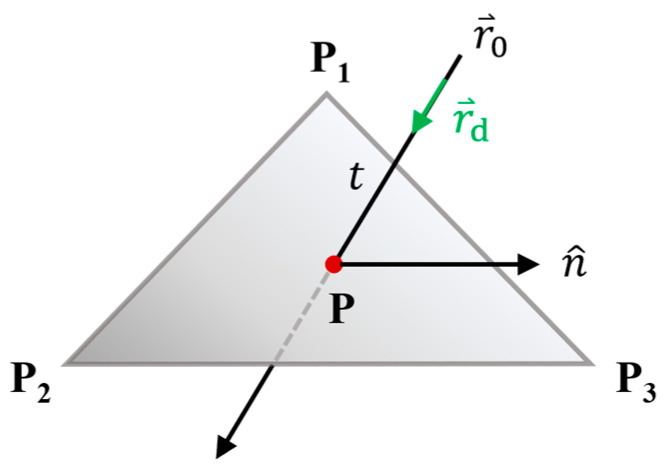
Ray–mesh intersection in 3D space using ray casting.

In this study, ray casting [37] was applied to calculate the entry and exit points (the motion path of the radiation beam), where rays intersect with organ surfaces, enabling identification of the traversed mesh regions. As illustrated in Figure 3, a ray is projected from an origin point ${\overset{⃑}{r}}_{0}$ in the direction of a vector ${\overset{⃑}{r}}_{d}$. The ray intersects the surface of a triangular mesh, whose surface normal is denoted by $\hat{n}$, and a point on the plane is represented as P. The intersection occurs at a distance t from the ray origin, and the intersection point is computed using the ray’s parametric equation (Equation (1)). The scalar t is determined by first formulating the plane equation (Equation (2)).

(1) $$P={\overset{⃑}{r}}_{0}+t{\;\cdot \;\overset{⃑}{r}}_{d},$$

(2) $$D=\hat{n}\;\cdot \;P,$$

From Equations (1) and (2), the ray-plane intersection condition gives:

(3) $$t=\frac{D-\hat{n}\;\cdot \;{\overset{⃑}{r}}_{0}}{\hat{n}\;\cdot \;{\overset{⃑}{r}}_{d}},$$

After solving for t, the intersection point P is calculated using Equation (1). To check if the intersection is inside the triangular mesh, a point-in-triangle test is performed. This test evaluates the directional consistency of the cross products of vectors from the intersection point to the triangle vertices. A point P is considered inside the triangle if the following condition holds:

(4) $$\left[\left(P-P_{i}\right)\times \left(P_{i-1}-P_{i}\right)\right]\;\cdot \;\left[\left(P-P_{i}\right)\times \left(P_{i+1}-P_{i}\right)\right]\leq 0,$$

where i denotes the index of a triangle vertex ($P_{i}$), this condition ensures that the point lies within the triangle boundaries by confirming the relative orientation of the formed vectors.

#### 2.2.2. Model-Based Dose Estimation

**Figure 4:**
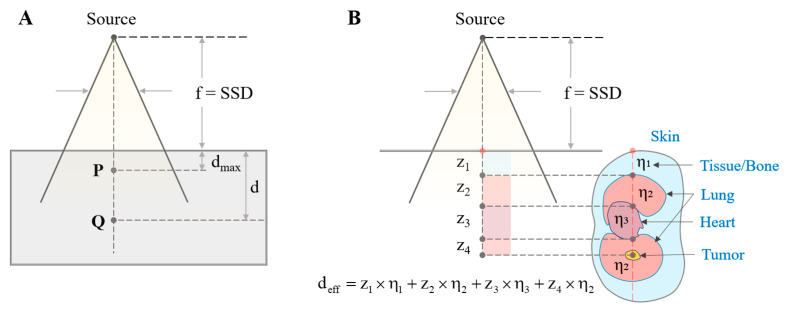
Schematic illustration of PDD measurement methods: (**A**) Standard PDD and (**B**) Equivalent depth-corrected PDD. (The figure illustrates a representative lateral beam incidence angle directed toward the patient body, passing through anatomical compartments with thicknesses $z_{i}$. Some components contributing to $d_{eff}$ may be omitted if the treatment beam does not traverse those regions at other irradiation angles).

PDD quantifies how the absorbed radiation dose varies with depth as a photon beam penetrates a medium. As illustrated in Figure 4, PDD is defined as the ratio of the absorbed dose $D_{Q}$ (at depth d) to the maximum absorbed dose $D_{P}$ (at depth $d_{max}$), expressed as Equation (5). The source-to-surface distance (SSD) is the distance between the radiation source and the surface of the medium. PDD values can be determined experimentally using a water phantom or tissue-equivalent material, or through mathematical modeling such as the Monte Carlo method.

(5) $$PDD=\frac{D_{Q}}{D_{P}}\times 100\%,$$

In this study, the buildup-tail method [38] was employed to simulate the absorbed dose at various depths. This method combines a quadratic buildup function in the form of $\frac{d}{\sqrt{d^{2}+n}}$ with an exponential attenuation tail $e^{-\mu d}$, as shown in Equation (6):

(6) $$PDD=\frac{d}{\sqrt{d^{2}+n}}\;\cdot \;e^{-\mu d},$$

where d is the depth in water (cm), n is the diffusion parameter (dimensionless), and μ is the attenuation coefficient (${cm}^{-1}$). By tuning n and μ, the PDD curve can be fitted for different photon energies.

Geometrically, the PDD curve is also influenced by the SSD. The dose at depth is governed by the inverse-square law, tissue attenuation, and scattering. The following Equations (7) and (8) describe PDD at two different SSDs, where $K_{s}$ is the scatter factor between d and $d_{max}$:

(7) $${PDD}_{1}=100\;\cdot \;{\left(\frac{{SSD}_{1}+d_{max}}{{SSD}_{1}+d}\right)}^{2}\;\cdot \;e^{-\mu (d-d_{max})}\;\cdot \;K_{s},$$

(8) $${PDD}_{2}=100\;\cdot \;{\left(\frac{{SSD}_{2}+d_{max}}{{SSD}_{2}+d}\right)}^{2}\;\cdot \;e^{-\mu (d-d_{max})}\;\cdot \;K_{s},$$

Combining Equations (7) and (8) gives the Mayneord Factor (F), which corrects the PDD for changes in SSD due to beam rotation:

(9) $$F={\left(\frac{{SSD}_{2}+d_{max}}{{SSD}_{1}+d_{max}}\right)}^{2}\;\cdot \;{\left(\frac{{SSD}_{1}+d}{{SSD}_{2}+d}\right)}^{2},$$

The relationship between the fluence in ion chamber in monitor units (MUs) and the absorbed dose in the patient (cGy) is expressed in Equation (10). In this equation, TD denotes the dose to be delivered at the tumor center, $D_{ref}$ represents the linear accelerator calibration dose (cGy/MU) at $d_{ref}$, $S_{c}\left(r_{c}\right)$ is the collimator scatter factor, $S_{p}\left(r\right)$ is the phantom scatter factor, and the SSD factor accounts for the distance correction, evaluated by Equation (11).

(10) $$TD={MU\times D}_{ref}\times \frac{PDD\left(SSD,r,d\right)}{100}\times S_{c}\left(r_{c}\right)\times S_{p}\left(r\right)\times SSD\;factor,$$

(11) $$SSD\;factor=\frac{{SSD}_{0}+d_{ref}}{SSD+d_{ref}},$$

where ${SSD}_{0}$ is the source-to-surface distance at which $D_{ref}$ is specified.

The lungs are located among inhomogeneous anatomical structures, each characterized by different attenuation coefficients. These tissue inhomogeneities can lead to significant deviations in dose estimations when using the standard PDD for homogeneous medium. To mitigate this limitation, a modified PDD that accounts for the heterogeneity of body tissues was employed. It is assumed that the effective thickness ($z_{eff}$) of an organ is equal to its actual physical thickness (z) scaled by a coefficient of equivalent attenuation relative to water (η), $z_{eff}=z\times \eta$. For a given material, η is also proportional to its relative electron density with respect to water. Based on this relationship, the dose at a point beyond an inhomogeneity can be determined based on the effective depth ($d_{eff}$) along the ray joining the point and the electron source, as illustrated in Figure 4B.

#### 2.2.3. Irradiated Volume Assessment

Normal tissue complication probability (NTCP) [39] is a widely used radiotherapy metric for evaluating the probability of radiation-induced side effects in healthy organs. It considers the absorbed dose, irradiated volume, and organ-specific radiosensitivity to predict the risk of complications. Standard NTCP estimation is commonly based on radiobiological dose–volume models, such as the Lyman–Kutcher–Burman (LKB) model and related formulations [40]. NTCP plays an important role in treatment planning by balancing effective tumor control with the minimization of harm to surrounding normal tissues, especially for radiation-sensitive organs such as the lungs or heart, where precise dose management is necessary.

The Effective Volume (${Vol}_{eff}$) in the LKB model [39] is a mathematical concept used to convert a complex, non-uniform dose distribution (like those found in IMRT or VMAT plans) into a simpler equivalent partial volume irradiation. This concept is rooted in the physiological architecture of the organ, specifically how its Functional Sub-Units (FSUs) are arranged. In the LKB NTCP model, this dependence is quantified by the parameter n. Understanding how tissue behaves differently to irradiation is critical for determining which cost functions and constraints should be prioritized during the inverse planning optimization process. For example, for n=1, the FSUs are arranged parallelly and are sensitive to the overall volume irradiated, demonstrating a pronounced volume effect. The formula transforms the DVH bins into a single effective volume [41]:

(12) $${Vol}_{eff}=\frac{1}{{Vol}_{ref}}\;\sum_{i=1}^{N}{Vol}_{i}\cdot {\left(\frac{D_{i}}{D_{max}}\right)}^{1/n},$$

To relate ${Vol}_{eff}$ to NTCP, an intermediate variable t is needed.

(13) $$t=\frac{D_{max}-{TD}_{50}({Vol}_{eff})}{m\cdot {TD}_{50}\left({Vol}_{eff}\right)},$$

In the preceding equation, $D_{max}$ is the maximal dose to the OAR. ${TD}_{50}$ is the dose at 50% probability of complication. Derived experimentally, m is inversely related to the dose–response curve.

(14) $${NTCP}_{\left(dose,\;volume\right)}=0.5+\frac{erf(\frac{t}{\sqrt{2}})}{2.0},$$

**Figure 5:**
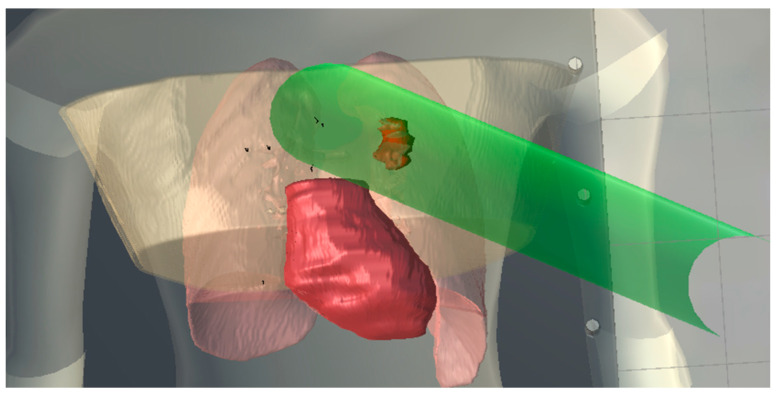
Illustration of a radiation beam intersecting a serial organ at risk.

Organs with a serial FSU, such as the spinal cord, brainstem, or optic chiasm, function as a chain in which the integrity of the entire organ depends on its weakest link. Damage to a small sub-volume in a serial FSU can lead to total functional failure. Serial tissues demonstrated little or no volume effect. The tolerance dose remains roughly the same whether a small section or the entire organ is irradiated. When optimizing treatment plans for serial organs, the primary objective must be to strictly limit the maximum point dose. The proposed framework enables identification of beam intersections with serial OARs, as illustrated in Figure 5.

**Figure 6:**
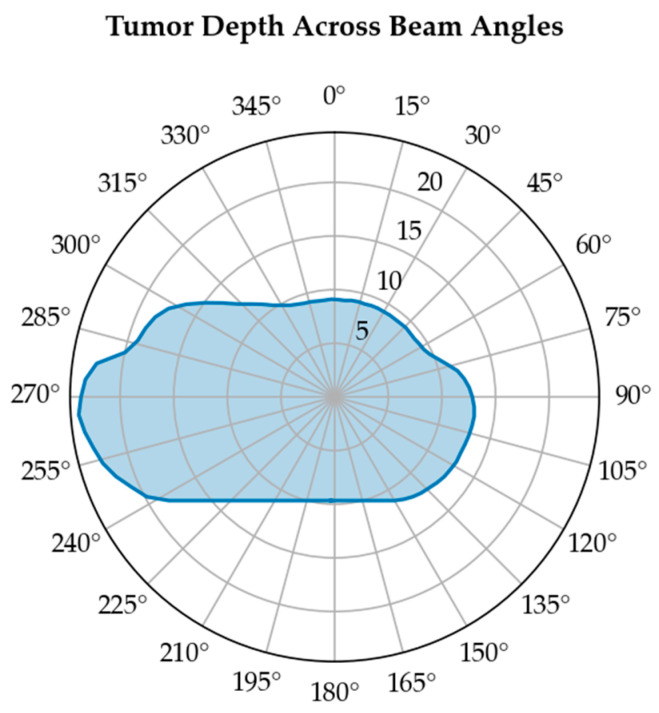
Tumor center depth (cm) across different beam angles for a tumor seated in the left lung.

In contrast, organs with a parallel architecture, such as the lung, liver, or parotid glands, possess a profound volume effect, meaning they can tolerate high doses to small sub-volumes, provided the rest of the organ is spared. The irradiated volume can be assessed by tumor depth across a range of gantry angles. Figure 6 shows how tumor depth varies with beam angle. The area under the curve provides a quantitative measure of the irradiated volume.

### 2.3. Radiation Therapy Simulation for Beam Arrangement and Collision Detection

**Figure 7:**
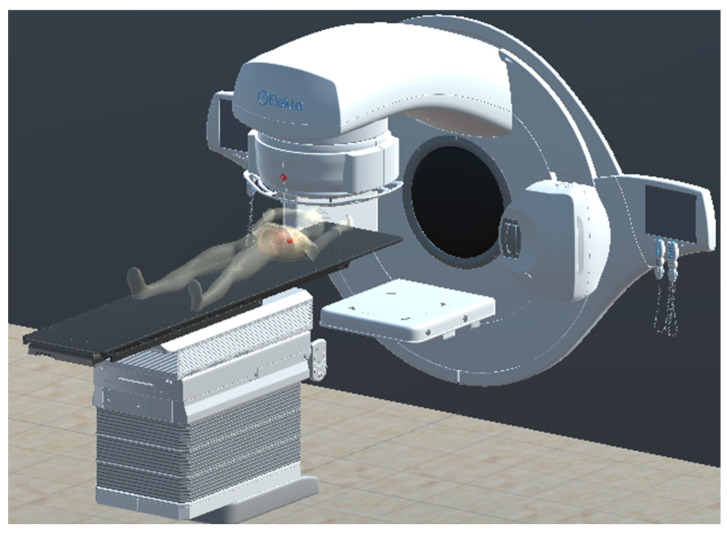
Radiotherapy simulation setup.

The simulation was developed in the Unity 3D environment using an institutional treatment unit (Elekta Versa HD), enabling the creation of a virtual radiotherapy scenario (Figure 7). The system provides two modes of operation. In the manual configuration mode, all treatment parameters are entered manually, allowing users to freely adjust beam arrangements and machine settings. In the RT plan mode, treatment parameters, including beam arrangements, dose rates, and collimator settings, are imported directly from an RT plan file, while patient table parameters are entered separately. In both modes, the system enables the detection of operational issues, such as collisions, and supports dose estimation throughout the treatment process. Consequently, it facilitates the selection and optimization of appropriate RT plans for effective treatment delivery.

The simulation was implemented on the Unity 3D platform to reconstruct realistic radiotherapy scenarios. A depth camera was used to capture point cloud data, while the structure and motion trajectories of the LINAC radiotherapy machine, along with the initial setup parameters, were obtained from manufacturer specifications and RT files. Spatial alignment between the treatment machine and the depth camera was achieved using the iterative closest point (ICP) algorithm combined with hand-eye calibration. Virtual markers from the camera were employed to assess and track discrepancies between planned and actual patient positions during treatment. The motion simulation and collision detection system was developed in our previous study [42], in which the separating axis theorem (SAT) algorithm was integrated with collision bodies defined by axis-aligned bounding boxes (AABB). Optimization techniques, including bounding volume hierarchy (BVH) and collision pair analysis, were implemented to reduce the computational complexity of collision detection and improve simulation efficiency.

## 3. Results

### 3.1. Comparison of Neural Network Recognition Performance

**Figure 8:**
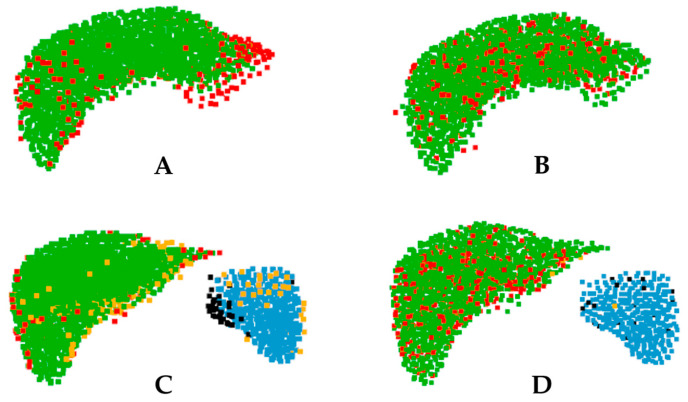
Segmentation results for: single-organ recognition, (**A**) Point Transformer and (**B**) PointNet++; and two-organ recognition, (**C**) Point Transformer and (**D**) PointNet++. (Color notation: Green—Liver; Blue—Spleen; Red—Liver prediction errors; Black—Spleen prediction errors; Yellow—Background prediction errors).

This study compares the performance of PointNet++ and Point Transformer (Seg50) for organ recognition using medical imaging data. As shown in Figure 8, the distribution of prediction errors differs between the two models. In the single-organ scenario (Figure 8A,B), the misclassifications of Point Transformer were mainly concentrated along organ boundaries, whereas PointNet++ exhibited a more random error distribution. In the two-organ scenario (Figure 8C,D), PointNet++ produced fewer spleen misclassifications.

Segmentation accuracy was evaluated using the Dice similarity coefficient (DSC), defined in Equation (15), where A represents the set of ground truth points and B the set of predicted points:

(15) $$Dice=\frac{2\left|A\cap B\right|}{\left|A\right|+\left|B\right|},$$

The comparison is summarized in Table 1. Both models were trained for 500 epochs on 100 training samples under identical conditions. Point Transformer completed training approximately 1 h faster than PointNet++. Inference speed was similar, with both models requiring about 1 s per case. For segmentation accuracy, Point Transformer outperformed PointNet++ in single-organ recognition, achieving a Dice score of 93.86 ± 1.50% compared to 91.80 ± 6.47% for PointNet++.

### 3.2. Post-Processing of the Segmented Organ Model

**Figure 9:**
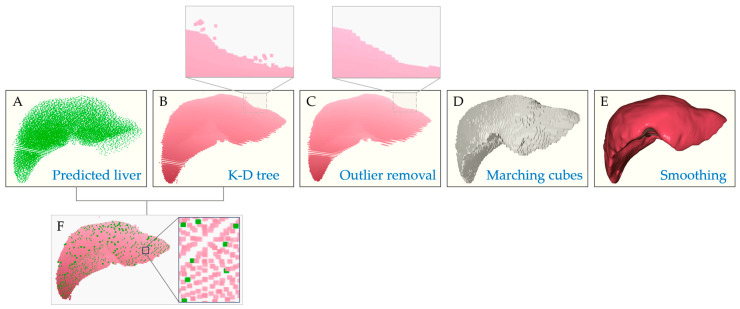
The process of 3D surface reconstruction from the Point Transformer-based predicted liver point cloud data to a complete 3D liver model for subsequent dose evaluation. The liver model undergoes the steps: (**A**) Predicted by the Point Transformer neural network. (**B**) Enhanced point cloud using a k-d tree. (**C**) Noisy boundary points removal using a statistical outlier removal algorithm. (**D**) Marching cubes. (**E**) Smoothed to refine the surface. (**F**) A mixed point cloud model showing the point density distribution of the predicted model and the post k-d tree model.

The results of processing the segmented organ point cloud data from the Point Transformer are summarized in Figure 9. K-d tree indexing of the predicted organ point cloud (Figure 9A) supports rapid spatial queries. For each point in the dataset, the 10 nearest neighbors were identified, and organ labels were assigned via majority voting among these neighboring points. This approach allowed the reconstruction of the original high-resolution point cloud, as illustrated in Figure 9B. Statistical outlier removal eliminates points whose distances to their 30 nearest neighbors exceed two standard deviations above the mean. It takes advantage of the logarithmic-time efficiency of the k-d tree and the effectiveness of outlier filtering, resulting in robust label propagation and enhanced segmentation quality (Figure 9C).

For mesh generation, the marching cubes algorithm was used to convert the labeled point cloud into a voxelized representation and reconstruct a continuous surface mesh. The voxel spacing was defined according to the original image resolution, with 0.97656 mm × 0.97656 mm in-plane resolution and 2.99 mm slice spacing, and the surface was extracted using a step size of 1. Triangular faces were generated by evaluating the local voxel configuration according to predefined surface-intersection patterns. This produced a continuous mesh that delineates the liver boundaries, as shown in Figure 9D.

The reconstructed mesh was further refined using Laplacian smoothing (Figure 9E). Specifically, smoothing was performed with 80 iterations and a relaxation factor of 0.1, whereby each vertex was iteratively adjusted toward the average position of its neighboring vertices to reduce surface irregularities while preserving the overall anatomical structure. After smoothing, only the largest connected mesh component was retained to remove isolated mesh fragments and preserve the principal organ structure. Together, these steps combine neighborhood-based label propagation, outlier filtering, voxel-based surface reconstruction, and mesh refinement to produce an organ model with improved accuracy and surface continuity, which is ideal for subsequent dose estimation.

### 3.3. Absorbed Dose Analysis

The depths and spatial locations of the anatomical structures along the beam path were determined using ray–mesh collision calculations in the ray casting algorithm. Tissue inhomogeneity was corrected using the equivalent PDD. The effective depth of the tumor, $d_{\mathrm{eff}}$, was obtained by summing the effective thicknesses, $z_{eff}$, of all tissue layers traversed by the beam (Figure 4B). For model simplification, soft tissue and bone were combined into a single layer with an attenuation equivalent to that of water ($\eta_{1}=1$) [20]. The density of the heart was referenced from Manning et al. [43] as 1.050 g/cm^3^, which is approximately equal to that of water ($\eta_{3}\approx 1$). The average density of lung tissue was taken as 0.275 g/cm^3^ ($\eta_{2}=0.275$) [44]. Using the PDD curves based on the buildup-tail function, the absorbed dose at the tumor center was calculated.

**Figure 10:**
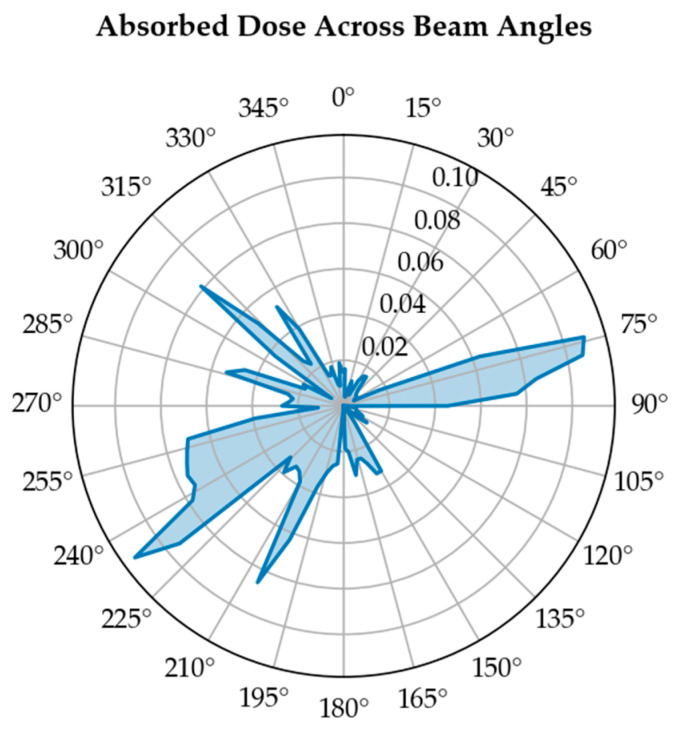
Distribution of absorbed dose (Gy) at tumor center across different beam angles in a VMAT treatment plan for left lung tumor.

Tumor dose evaluation at different depths and beam angles ensures adequate target irradiation while minimizing NTCP in surrounding normal tissues. As shown in Figure 10, the absorbed dose shows a clear dependence on beam angle. The spatial relationship between the beam path and surrounding organs strongly affects the dose distribution. Certain beam directions result in higher dose deposition in sensitive structures, while others maintain lower exposure levels.

To validate the accuracy of the proposed model, the calculated absorbed doses were compared with ground-truth values obtained from a clinical RT plan at Chiayi Christian Hospital. The reference plan was generated using Pinnacle, a high-accuracy TPS with a reported dose error within 2% [45]. Besides the inhomogeneous case of the lung, two homogeneous organs, namely the liver and the brain, were included for comparison. As shown in Table 2, the dose discrepancy between the proposed model and Pinnacle is 13.8% for the lung case, while it is only 5.9% and 3.5% for the liver and brain, respectively. These findings indicate that tissue inhomogeneity, where density and elemental composition differ from water or soft tissue, can markedly affect the accuracy of correction-based methods such as PDD. Even with equivalent-depth PDD, the inhomogeneous organ showed a noticeably lower accuracy compared to the homogeneous cases.

### 3.4. Evaluation of Irradiated Volume

**Figure 11:**
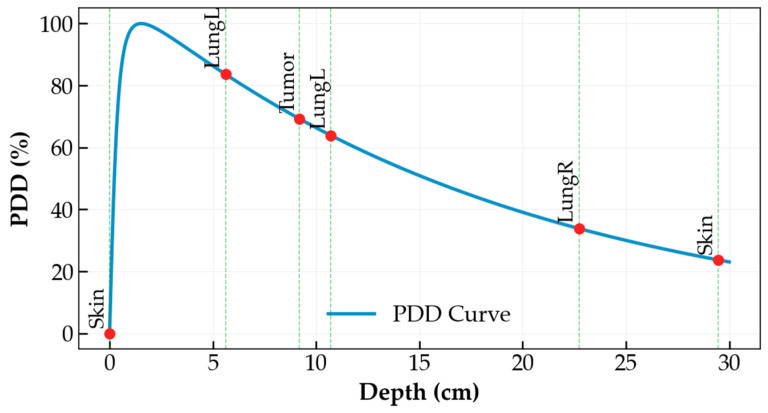
Evaluation of the beam arrangement for a pulmonary tumor.

Reducing the irradiated volume is the most effective strategy for improving treatment plan quality and ensuring patient safety for parallel organs like the lung, liver, and kidney. The radiobiological rule of thumb for parallel organs is that delivering a high dose to a small sub-volume is far safer than distributing a moderate or low dose across the entire organ. Ray casting provides an effective method for determining tumor depth from the organ surface across different beam angles. The result is illustrated in Figure 11, which shows a PDD profile along a representative ray path, with depths corresponding to the skin, lung, and tumor locations.

### 3.5. Simulation of Treatment Delivery

The developed simulation system supports two modes of operation. In the manual configuration mode, all parameters are specified manually to optimize the beam arrangement. In the RT plan mode, beam paths and prescribed doses are imported from a treatment planning file, while patient table parameters are entered manually. The interface displays both the machine-delivered dose in MU and the corresponding tumor absorbed dose in Gy.

**Figure 12:**
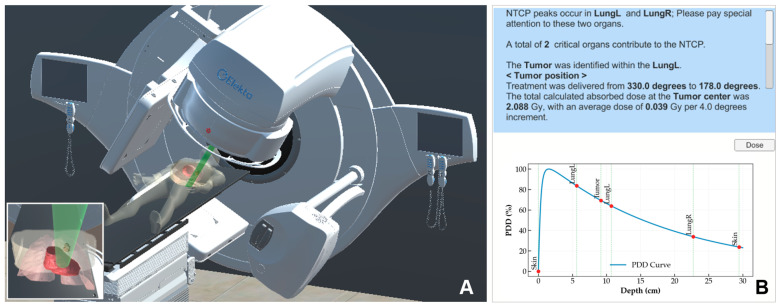
(**A**) Visualization of radiotherapy treatment setup, and (**B**) corresponding absorbed dose at the tumor site.

Post-processed organ models, the phantom, and the machine model were used to construct the essential components of the radiation therapy dose simulation. All models were scaled and aligned to match the dimensions of clinical equipment, enhancing realism (Figure 7). The control interface features a simulation panel for configuring beam output, beam size, and machine-couch positioning. RT plan files can be loaded to import machine trajectory data, enabling the selection of specific treatment paths. When a treatment path simulation is executed, the system visualizes beam–organ interactions and verifies tumor targeting accuracy. Integrated dose estimation functions generate a report showing the applied dose and tumor absorbed dose results for each beam angle (Figure 12).

**Figure 13:**
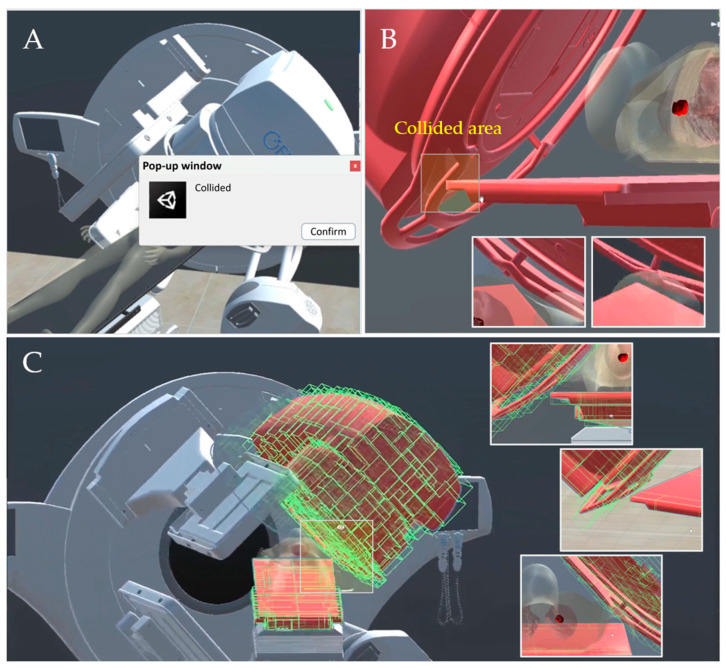
Collision detection function: (**A**) Pop-up collision alert window; (**B**) collided area between couch and gantry components from the user’s perspective; (**C**) from the developer’s perspective, the collision verified by the intersection of the AABBs.

The collision detection feature was implemented using motion data extracted from RT plan files and aligned with real-world measurements. When a collision occurs, the system triggers an alert through a pop-up window and highlights the collided parts to help users identify the issue (Figure 13A). This system demonstrated reliable performance in identifying potential machine-patient and machine-machine interactions within the predefined 5 cm safety margin. In our previous validation study [42], 10 real-world treatment scenarios, including both coplanar and noncoplanar arrangements, were all correctly identified by the system, corresponding to a detection rate of 100% in this test set. In addition, optimization using bounding volume hierarchy (BVH) and collision pair analysis substantially improved computational efficiency by reducing the number of bounding volumes by more than 95% for the radiation delivery and imaging system and more than 99% for the patient positioning system.

Due to the AABB-based SAT approach, collisions may appear as small gaps from the user’s perspective (Figure 13B). However, the intersections of the AABBs can still be verified from the developer’s perspective for collision prediction (Figure 13C). Therefore, the occasional conservative false-positive detections may appear visually as small gaps between components. Nonetheless, these remained within the clinically acceptable safety margin and represent a practical trade-off between spatial accuracy and real-time simulation speed.

## 4. Discussion

This study presents a Unity 3D-based radiotherapy simulation platform integrating AI-driven organ segmentation, model-based dose estimation, and collision detection. Point Transformer achieved over 90% segmentation accuracy, while post-processing improved anatomical fidelity for dose estimation. Using a clinically oriented left lung tumor example, patient CT-derived anatomy was automatically segmented and reconstructed into refined 3D organ models. The resulting models were imported into the simulation platform. Candidate beam directions were then evaluated based on tumor depth, beam-path dose estimation, irradiated-volume effects in parallel and serial organs, and collision risk during treatment delivery. The platform therefore enhanced treatment visualization and supported collision risk assessment. It also provided relevant dose metrics and served as an interpretable pre-planning environment for early feasibility assessment, beam-angle screening, and safety evaluation before full dosimetric optimization. However, it was intended to complement rather than replace a commercial TPS.

The proposed Point Transformer model achieved markedly higher segmentation accuracy than the traditional PointNet++ baseline. In single-organ liver segmentation, it reached a DSC of 93.86 ± 1.50% versus 91.80 ± 6.47% for PointNet++ (Table 1), reflecting its ability to capture both local and global geometric features through self-attention for precise and consistent contours. This result is consistent with broader point cloud benchmarks, where transformer-based networks outperform earlier point-based models by several percentage points in segmentation accuracy [15]. As shown in Figure 8, most Point Transformer errors occurred near organ boundaries, while PointNet++ misclassifications were more randomly distributed, indicating the transformer produced more coherent and anatomically faithful segmentations. In multi-organ segmentation (liver and spleen), the transformer still outperformed PointNet++ (91.86 ± 3.25% vs. 90.79 ± 5.78% Dice), though performance dropped slightly compared to the single-organ case, likely due to class overlap or smaller organ size. This process may introduce minor errors in the geometric representation of organs, which could subsequently affect the accuracy of dose estimation. This study presents a proof-of-concept framework for CT-based point-cloud segmentation, currently demonstrated for the liver and spleen. In addition, due to hardware limitations, the model was trained using only 32,000 points per case. Nevertheless, the proposed framework can be extended and adapted to newer and more efficient models in future work. Although the same approach may be applied to other OARs, further validation across a wider range of anatomical regions will be necessary to confirm its generalizability.

In addition to the segmentation, the absorbed dose analysis further demonstrates the clinical relevance of the proposed work. The system used ray-casting-based depth estimation with equivalent-depth PDD correction to generate dose distributions. For homogeneous organs such as the liver and brain, these distributions aligned well with those produced by a commercial TPS, with deviations below 6%. However, inhomogeneous tissues such as the lung showed a larger discrepancy (13.8%). Unlike convolution/superposition or Monte Carlo approaches, PDD does not explicitly model scatter, lateral electron transport, or complex heterogeneous media, and therefore its accuracy is reduced in highly inhomogeneous regions. Furthermore, geometric errors resulting from automated segmentation also contributed to the final accuracy, as previously characterized by Van Herk et al. [46]. While analytical PDD-based correction provides a reasonable estimation for rapid dose assessment, further integration of advanced physics-based methods (e.g., Monte Carlo simulation or convolution-superposition algorithms) may be required to reduce uncertainty in aerated structures and improve the calculation accuracy, especially in heterogeneous tissues. Nevertheless, the proposed platform provides a flexible simulation framework that could be extended in future studies to incorporate more advanced physics-based dose calculation algorithms. However, due to high computational demands, convolution/superposition and Monte Carlo methods would more appropriately be implemented as native back-end solvers rather than as fully in-Unity C# routines, as in the current workflow.

Compared to commercial TPS such as RayStation and Varian Eclipse [26], our platform emphasizes transparency, cost-efficiency, and customization. Commercial TPS offers advanced features, but relies on proprietary black-box algorithms tied to specific hardware and service contracts, limiting adaptability and driving high costs. In contrast, the proposed framework offers transparency and flexibility, enabling users to inspect, validate, and customize segmentation, dose estimation, and collision detection modules. This flexibility facilitates the integration of new AI models, alternative dose estimation methods, and institution-specific constraints without reliance on commercial licensing or hardware-specific platforms. The Unity-based virtual environment supports intuitive visualization and training, allowing systematic evaluation of beam–organ interactions, dosimetric effects, and collision risks. From a clinical workflow perspective, the proposed system is positioned as a supplementary clinical decision-support tool for beam-angle optimization, preliminary feasibility assessments, and safety evaluations. The proposed framework offers advantages in accessibility, customization, and workflow integration, with the potential to improve efficiency and cost-effectiveness. However, formal quantitative evaluation of these benefits compared with existing systems remains for future study. While not intended to replace commercial TPS for final dose prescription or definitive plan verification, it provides an extensible, low-cost platform for dosimetric analysis and educational applications in radiation oncology.

## 5. Conclusions

The proposed integrated radiotherapy simulation system successfully combines advanced AI-based segmentation, dose estimation, and collision detection within a unified virtual planning environment. Using a Point Transformer architecture with point-cloud processing and post-processing refinements, the system achieved acceptable segmentation accuracy, enabling precise delineation of OARs. Ray casting was employed for radiation dose computation, while collision detection in Unity 3D improved treatment safety and planning efficiency. These results highlight the potential of the system to streamline clinical workflows, enhance anatomical fidelity, and support more accurate, patient-specific radiotherapy strategies through a transparent and low-cost implementation.

## Figures and Tables

**Table 1:** Performance comparison of Point Transformer and PointNet++.

Case	Method	Total Epoch	Training Data	Training Time	Prediction Time	Prediction Dice Score
Single-organ	Point Transformer v1	500	100	~9.5 h	<1 s	93.86 ± 1.50%
	PointNet++	500	100	~10.5 h	~1 s	91.80 ± 6.47%
Two-organ	Point Transformer v1	500	100	~9.5 h	<1 s	91.86 ± 3.25%
	PointNet++	500	100	~10.5 h	~1 s	90.79 ± 5.78%

**Table 2:** Dose comparison at tumor centers for three patients between the equivalent depth model and Pinnacle TPS.

Tumor Location	Equivalent Depth Model (Gy)	Pinnacle (Gy)	Difference (%)
Lung	8.84	7.77	13.8
Liver	5.12	5.44	5.9
Brain	19.78	19.12	3.5

## Data Availability

The original contributions presented in this study are included in the article. Further inquiries can be directed to the corresponding author.
